# Suicide resilience: A concept analysis

**DOI:** 10.3389/fpsyt.2022.984922

**Published:** 2022-09-26

**Authors:** Xinlu Wang, Zhongqiu Lu, Chaoqun Dong

**Affiliations:** ^1^School of Nursing, Wenzhou Medical University, Wenzhou, China; ^2^Emergency Intensive Care Unit, Emergency Department, The First Affiliated Hospital of Wenzhou Medical University, Wenzhou, China

**Keywords:** suicide resilience, suicide prevention, protective factors, mental health, concept analysis

## Abstract

**Objective:**

Suicide resilience is gaining increasing attention from researchers because of its potential role in preventing suicide. However, it has not been clearly analyzed, and there are various meanings and terms regarding this issue. The purpose of this analysis, therefore, was to conceptualize the concept of suicide resilience.

**Methods:**

Walker and Avant's method of concept analysis was used to identify the antecedents, attributes, and consequences of suicide resilience. The literature was searched using PubMed, PsychINFO, Embase, Web of Science, CINAHL, CNKI, and WanFang databases with no limitation on publication date. The search included peer-reviewed journal articles and dissertations related to suicide resilience published in English or Chinese.

**Results:**

52 articles were identified to provide information for this concept analysis. Five defining attributes of suicide resilience were identified: social support, coping strategies, psychological capital, meaning in life, and sense of responsibility. Antecedents of suicide resilience were high suicide risk events, and consequences of suicide resilience were keeping vulnerable individuals stay away from or overcoming suicidality.

**Conclusions:**

The result of the analysis provided a more clear definition of suicide resilience. The identified defining attributes, antecedents, and consequences can be further tested and used to develop potential interventions. Future research is needed and will help to advance our understanding of the scope.

## Introduction

Suicide is a leading cause of death worldwide, presenting a significant public health issue (1). According to the World Health Organization (WHO), over 700,000 individuals die by suicide annually. It is estimated that for each suicide death, there are many more individuals who attempt suicide or have serious thoughts about taking their lives (2). Given the far-reaching harmful effects of suicide on individuals, families, and societies, it is crucial to advance suicide prevention. Currently, research on suicide prevention has mainly focused on the risk factors that increase an individual's vulnerability to suicide, and a bunch of these factors such as psychiatric disorders, suicidal ideation, and prior suicide attempts, have been identified (3, 4). These studies enabled us to gain a much deeper understanding of suicide and find vulnerable individuals timely. However, several researchers have indicated that the predictive validity of these risk factors is limited, and a sole focus on suicide risk factors is not enough for suicide prevention strategies (5). Recently, there has been a shift toward additional consideration of factors that confer resilience against suicide (6).

Resilience is an emerging concept in suicide research. The notion was derived both from ecology and psychology, and used by various disciplines (7). Although it has different meanings in different contexts, resilience generally refers to the ability, outcome, or dynamic process of overcoming adversity adaptively (8). With the observation of the fact that a number of individuals exposed to high suicide risks would not be suicidal, researchers in the suicide field have become more and more interested in the phenomenon of resilience (9). Empirical findings have indicated that resilience represents an intermediate between suicide risk factors and suicidality, and may mitigate the adverse effect of suicide risk factors (10, 11). Furthermore, individuals with low resilience were associated with an increased risk of lifelong suicidal behaviors (12). Thus, resilience is increasingly recognized as a suicide protective factor. In line with the broader research, resilience in suicide has also focused on identifying factors that can facilitate resilience, and a repertoire of potential factors from general resilience studies has been found (13). Nevertheless, it is suggested that some of these factors, such as positive attributional style and meaning in life, exhibit better protective qualities in the specific context of a suicidal crisis (14, 15). Meanwhile, a cluster of these factors shows better predictive validity of who can be resilient to suicide risks (16). Hence, to better understand and investigate resilience in suicide, researchers have termed it suicide resilience (17).

While there is a growing body of work on suicide resilience, the concept remains ambiguous. Currently, there are several inconsistent definitions of the term. For example, Osman defines suicide resilience as the perceived ability, resources, or competence to regulate suicide-related thoughts, feelings, and attitudes (17); whereas Johnson asserts that suicide resilience is a perception or set of beliefs that buffer individuals from suicidality in the face of stressors (14). Moreover, the process view of general resilience has dramatically influenced the definition of suicide resilience. Some scholars investigated suicide resilience as a dynamic process and used the term such as suicidal recovery to describe it (18). Additionally, there are several other phrases for suicide resilience, such as resilience to suicidality, psychological resilience to suicidal experience, and overcome suicidality (14, 19, 20). Due to these existing fuzzy interpretations, the recent increase in literature on suicide resilience may cause more confusion than clarity among researchers and clinicians, impeding precise translation of the concept into practice.

It is increasingly acknowledged that resilience is a promising intervention target for individuals facing adversity, including suicidal risk. Few researchers have developed and implemented resilience-oriented programs, indicating that they can reduce participants' suicide risk (21, 22). However, all of the programs targeted promoting general resilience, rather than suicide resilience. This may be because the suicide resilience concept is still unclear and our knowledge of suicide resiliency factors is limited. Although existing interventions have been proven to be beneficial, interventions specifically designed for suicide resilience are believed to be more effective for those in a suicidal crisis (14). Consequently, there is a dearth of research to clarify the concept of suicide resilience to further improve existing suicide prevention strategies.

In this study, we analyzed the concept of suicide resilience to address the above-mentioned research gap using Walker and Avant's concept analysis framework. Although there are various approaches for concept analysis, Walker and Avant's method is the most widely used one (23). Numerous researchers, particularly in nursing, have employed this method to examine the basic elements of health-related concepts, such as digital resilience, perinatal resilience, and vaccine literacy (24–26). Through its systematic steps, we expect to provide clarity for researchers and clinicians about suicide resilience and stimulate more research on the issue.

## Methods

### Concept analysis method

Walker and Avant's method of concept analysis was used. It involves eight iterative steps to guide a deeper understanding of a concept. The eight steps include selecting a concept, determining the aims of analysis, identifying all uses of the concept, determining the defining attributes, identifying a model case, identifying additional cases, identifying antecedents and consequences, and defining empirical referents (27). Adaptation of these steps in the study was shown in Table 1.

**Table 1:** Adaptation of Walker and Avant steps of concept analysis.

**Steps of concept analysis**	**Description of the steps in this study**
1. Select a concept	Suicide resilience was selected as the concept for this analysis
2. Determine the aims of analysis	This analysis aimed to conceptualize suicide resilience
3. Identify all uses of the concept	Identify possible uses of the concept of suicide resilience from various resources such as dictionaries and research databases
4. Determine the defining attributes	Determine the characteristics that are most frequently associated with the concept of suicide resilience
5. Identify a model case	Identify an example of suicide resilience that contains all the defining attributes
6. Identify additional cases	Two additional cases (borderline and contrary cases) were identified in this analysis. A borderline case is an instance that contains most (but not all) defining attributes of suicide resilience. A contrary case is an instance that contains none of the attributes
7. Identify antecedents and consequences	Identify the events that take place prior to the occurrence of suicide resilience, and those occur as an outcome of the appearance of suicide resilience
8. Define empirical referents	Define the empirical referents that measure the existence and attributes of suicide resilience

### Data source

A systematic search was conducted in online databases, including PubMed, PsychINFO, Embase, Web of Science, CINAHL, CNKI (Chinese), and Wanfang (Chinese) on Aug 30, 2022. We used the following terms in each database: “suicid^*^,” “self-harm,” “self-injur^*^,” “self-destruct^*^,” “self-inflict^*^,” “self-mutilat^*^,” “overdos^*^,” “self-poison^*^,” “resilien^*^,” “hardiness,” “buffer^*^,” “bounce back,” “recover^*^,” “resist^*^,” “rebound,” “adapt^*^,” “overcome^*^.” The online databases were searched for titles, abstracts, or topics/subjects containing these terms. The Boolean operator “OR” was used to connect the similar concept, and “AND” was used to combine diversity. No date limitation was applied in the search strategy. The search strings in each database are presented in Supplementary material. In addition, the reference lists of relevant literature were searched manually to reduce the risk of missing potentially important research.

### Data selection

**Figure 1:**
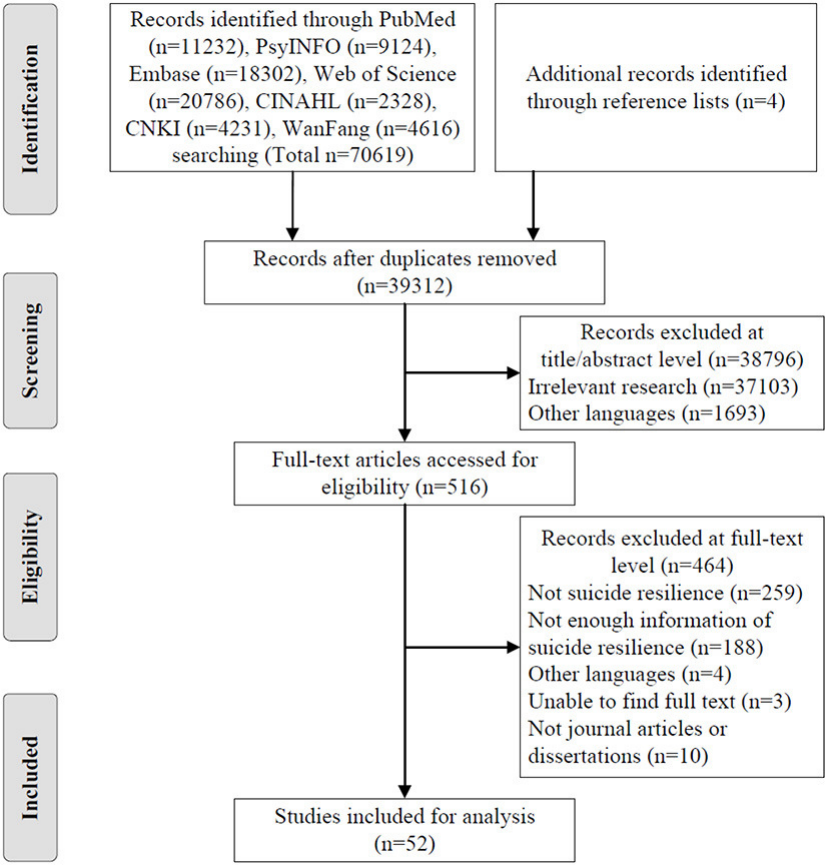
PRISMA literature search flowchart.

The inclusion criteria for selecting relevant articles were: (i) discussing or investigating resilience specifically to suicidality; (ii) referring to at least one of the following items of suicide resilience: definition, attributes, antecedents, consequences, or measurement techniques; (iii) peer-reviewed journal articles, or dissertations in English or Chinese. The exclusion criteria were: (i) only focusing on general resilience that is unrelated to suicide; (ii) focusing on resilience beyond the level of the individual (e.g., family resilience). Of the original 70,619 studies that were found, 39,312 studies remained after excluding duplicates as potential eligible citations. Based on the title and abstract screening level, 38,796 studies were removed, which was obviously against eligibility criteria. Afterward, we thoroughly read the full text of the remaining 516 studies and included 52 articles that were particularly relevant to this analysis. Figure 1 is the PRISMA flowchart of the whole selection process.

### Data analysis

To analyze data, we read each article in detail and extracted the following information from them: detailed characteristics, study sample, attributes, antecedents, consequences, and measurements of suicide resilience (Table 2). Additionally, all listed attributes were put together in another file to more clearly find the characteristics frequently relevant to the concept of suicide resilience. In this separate file, we regrouped synonymous and closely related attributes into the same theme. We then counted the number of articles that mentioned the theme and selected the top-mentioned themes for further analysis. The selection of attributes was discussed and reviewed by all authors throughout.

**Table 2:** Characteristics of the included literature.

**No**.	**References, country**	**Design**	**Sample**	**Antecedents**	**Defining attributes**	**Consequences**	**Empirical referents**
1.	Fenaughty et al. (28), New Zealand	Qualitative	Young gay men	The coming-out process	Social support, high self-esteem, active efforts to develop effective coping strategies	Swing the finely balanced seesaw away from a suicide attempt	Interview
2.	Osman et al. (17), USA	Quantitative	Adolescents and adults	Suicide risk	Internal protection, external protection, emotional stability	Reduce suicide risk	Suicide Resilience Inventory
3.	Everall et al. (29), Canada	Qualitative	Previously suicidal female	Being suicidal	Social support, emotion-focused coping strategies, shifting to positive perspective, purposeful and goal-directed action	Overcome suicidality	Interview
4.	Bostik (30), Canada	Dissertation (Qualitative)	Those who had experienced adolescent suicidality	Being suicidal	Supportive relationships, feeling safe, willingness to turn to others for help and support, finding hope, sense of control, personal responsibility, positive thinking, meaning in life, thinking about others	Able to cope effectively with feelings of despair, better equipped to handle future challenges successfully	Interview
5.	Lakeman et al. (31), Australian	Review	Those who had experienced suicidal ideation	Being suicidal	Connection with others	Live with or get over being suicidal	N/A
6.	Bergmans et al. (32), Canada	Qualitative	Young adults between the ages of 18–25 years, who have a history of two or more suicide attempts	Repeated suicide-related behavior	Becoming aware of choices, diagnostic education, becoming aware of emotions, understanding the role of emotion as part of human experience, and learning to identify and tolerate emotions	Transition from higher to lower risk of suicide, moving from “living to die” to “dying to live”	Interview
7.	Johnson et al. (33), UK	Quantitative	Participants with schizophrenia-spectrum disorders	Schizophrenia-spectrum disorders	Positive self-appraisals (particular appraisals of emotion coping ability)	Buffer the pernicious impact of hopelessness in the development of suicidal thoughts	Resilience Appraisals Scale
8.	Johnson et al. (34), UK	Quantitative	College student	Stressful life events	Positive self-appraisals	Buffer the effect that negative life events have on suicidality	Resilience Appraisals Scale
9.	Johnson et al. (14), UK	Review	N/A	Suicide risk	Positive attributional style, high levels of agency, problem-solving ability, self-esteem, problem-solving confidence, social support	Buffer individuals from the development of suicidality in the face of risk factors or stressors	N/A
10.	O'Dwyer et al. (35), Australian	Qualitative	Family carers of people with dementia	Intense and demanding work of caring for a family member with dementia	Coping strategies, personal characteristics such as flexibility, determination and compassion, social support, faith	Do not experience suicidal thoughts, despite challenging care situations, or have refrained from acting on suicidal thoughts	Interview
11.	Sun et al. (18), Taiwan	Qualitative	Patients who recovered from suicide attempts and their caregivers	Suicide attempt	Self-reflection and to live for family, strong support systems, becoming flexible and open-minded, rebuild a positive sense of self, positive coping strategies to deal with stress, self-care	Keep going to achieve a satisfying life	Interview
12.	Kleiman et al. (15), USA	Quantitative	Undergraduate students	Suicide risk	Meaning in life	Decrease suicidal ideation over time and decrease lifetime odds of a suicide attempt	N/A
13.	Kleiman et al. (36), USA	Quantitative	College students	Suicide risk	Meaning in life, gratitude, grit	Decline suicidal ideation over time	N/A
14.	Benson (37), USA	Dissertation (Quantitative)	Veterans	Combat distress	Sense of coherence (the ability to understand, manage, and find meaning in negative life experiences)	Successfully cope with their combat experiences and prevent subsequent suicidality despite how traumatic they perceive their experiences to be	Suicide Resilience Inventory
15.	Chi et al. (38), Taiwan	Qualitative	People who had attempted suicide	Suicide attempt	Self-awareness, inter-relatedness of life, coping in a healthy manner with the stresses in life, acceptance of self, others, and of life itself, adjusting to the reality	Regain the desire to be alive and begin investing in life	Interview
16.	Reading et al. (20), UK	Qualitative	Prisoners with a past experience of suicidal thoughts, feelings, or attempts	Being suicidal	Sense of self, presence of meaning, connectedness, shift of perspective, and re-establishing control	Overcome suicidality	Interview
17.	Panagioti et al. (39), UK	Quantitative	Individuals who had previously been exposed to a traumatic event and reported PTSD symptoms in the past month	PTSD symptoms	Perceived social support	Buffer individuals with PTSD symptoms against the development of suicidal thoughts and behaviors	N/A
18.	Kleiman et al. (40), USA	Quantitative	College students	Suicide risk	Social support and positive events	Buffer the relationship between negative events and suicidal ideation	N/A
19.	Matel-Anderson et al. (41), USA	Qualitative	Nurses who had experiences working with adolescent inpatients admitted for a suicide attempt	Suicide attempt	Connection with others, future plans, faith or a belief, expression of feelings, and communicating stressful thoughts	Prevent future suicide attempts and completion	Interview
20.	Heisel et al. (42), Canada	Quantitative	Community-residing older adults over 65 years old	Late-life suicide risk	Meaning in life	Protect against the onset or exacerbation of late-life suicide ideation	N/A
21.	Chan et al. (43), Canada	Qualitative	Participants who have contemplated death by suicide	Being suicidal	Social support, religion and spirituality, reconnecting with self, realizing the impact on family, things to be done, goals for the future, healthy coping behaviors	Find reasons to go on living	Interview
22.	Kapoor et al. (44), USA	Quantitative	Low-income African American women who reported a suicide attempt and exposure to intimate partner violence in the prior year or currently	Childhood abuse, suicide attempt	Intrapersonal strength, self-efficacy, spiritual well-being, Internal protection, external protection, emotional stability	Protect an individual from engaging in suicidal behavior in response to major stressors, other risk factors, and suicidal thoughts	Suicide Resilience Inventory
23.	Crona et al. (45), Sweden	Qualitative	Inpatients diagnosed with severe depression who had attempted suicide	Being suicidal	Regaining control, relief in the personal situation, professional care	Make a decision to continue living	Interview
24.	Sellin et al. (46), Sweden	Qualitative	Participants who were admitted to psychiatric inpatient care related to suicide risk	Suicide risk	Reconnecting with oneself, expressing oneself, supportive relatives, professional care	Be capable of managing their own lives	Interview
25.	Sun et al. (47), Taiwan	Quantitative	People who had attempted suicide	Suicide attempt	Self-awareness of the value of life, application of coping strategies, striving to live a normal and satisfying life, better economic conditions, less frequent suicidal behavior	Create a more stable and fulfilling life, have an improved recovery from suicide	Suicidal recovery ability Scale
26.	Tofthagen et al. (48), Norway	Qualitative	People who have committed no self-harm during the past 2 years and experienced recovery from self-harm	Suicidal behavior	Stable relationship, inner pain expression, reconciling with oneself, engaging in alternative actions to self-harm, taking responsibility for themselves, receiving guidance from mental health nurse	Learn to choose life and cope with everyday life without the need for self-harm, gain a greater understanding of how one's own wellbeing can be promoted even without being completely ‘cured' of the illness	Interview
27.	Collins et al. (49), Australian	Quantitative	University students	Suicide risk	Mindfulness, zest for life	Lower levels of suicidal desire	N/A
28.	Siegmann et al. (50), German	Quantitative	University students	Depressive symptoms	Positive mental health, life satisfaction, social support	Buffer the impact of depressive symptoms on suicide ideation	N/A
29.	Sun et al. (51), Taiwan	Quantitative	Individuals who have attempted suicide	Suicide attempt	Self-awareness of the value of life, application of coping strategies, striving to live a normal and satisfying life	Reawaken hope and regain the desire to live	Suicidal Recovery Ability Scale
30.	Gallagher et al. (52), USA	Review	High-risk youth	High suicide risk	Individual assets such as problem-solving, cognitive style, self-esteem, ecological resources such as social support, meaningful activities	Reduce suicide risk	N/A
31.	Roberts (53), USA	Dissertation (qualitative)	Adults who thought about taking their own lives as an adolescent and decided against it	Being suicidal	Connectedness, hope, and love	Lessen the intensity of emotional pain, and not act on arising suicidal thoughts	Interview
32.	Shaw et al. (54), USA	Qualitative	Alaska Native or American Indian who had a self-reported history of seeking help for suicidality	Being suicidal	Positive social connections, responsibility, access to health services, skills to mitigate stress	Lessen the severity of their suicidality, stay safe during times when they had experienced thoughts of suicide, reduce suicide risk	Interview
33.	Matel-Anderson et al. (55), USA	Quantitative	College students 18 to 24 years old	Suicide risk	Social support, positive thinking, self-esteem	Decrease the risk of suicide	Suicide Resilience Inventory
34.	Gulbas et al. (56), USA	Qualitative	Latina adolescents who attempt suicide	Suicide attempt	Reconnection to family, access to mental health services, development of cognitive strengths and coping skills	Promote and sustain wellbeing following a suicide attempt, a reduction in feelings of hopelessness and suicidal thoughts	Interview
35.	Fullerton et al. (57), USA	Quantitative	Public school students in grades 9 to 12	Suicide risk	Positive adult relationships	Greatly reduce the odds of suicide attempt	N/A
36.	Harris et al. (58), UK	Qualitative	Individuals with non-affective psychosis or schizophrenia diagnoses who had the experience suicidal thoughts and behaviors	A diagnosis of schizophrenia and being suicidal	Understanding experiences, reasons to live, sense of security, responsibility to others, a desire for personal development, active behaviors including talking to people, keeping occupied, and feeling supported	Manage psychosis and the concomitant suicidal thoughts and behaviors	Interview
37.	Zaheer et al. (59), Canada	Qualitative	Chinese-born women living in Canada with a history of suicidal behavior	Being suicidal	Support from health professionals, family, and friends, spiritual support, self-care, creating goals for the future, and a sense of mastery	Reduce suicide ideation, and improve their ability to cope with stress and pressure	Interview
38.	Fuller-Thomson et al. (60), Canada	Quantitative	Canadians in chronic and disabling pain who had ever had serious suicidal thoughts	Suicide ideation relating to chronic pain	Be older, white, women, better educated, with a confidant and more likely to use spirituality or religion to cope, no history of mental health illness	Free of suicidal thoughts in the preceding year	N/A
39.	Wadhwa et al. (61), Canada	Quantitative	Three studies of suicide ideation among older adults	Late-life suicide risk	Reasons for living	Protect against suicide risk in later life	The Reasons for Living-Suicide Resiliency Scale
40.	Chen et al. (62), Taiwan	Dissertation (qualitative)	Adult suicide attempters	Suicide attempt	Willing to seek help, healthy coping behaviors, strengthening ability and confidence to face and solve problems, adjust thinking, social support, religion, health professionals, responsibility	Move away from the suicide crisis, and move toward life adaptation and recovery	Interview
41.	Clement et al. (63), USA	Quantitative	College students	Suicide risk	Optimism, hope, and grit	Reduce suicide risk	N/A
42.	Harris et al. (64), UK	Review	N/A	Schizophrenia diagnoses	Perceived social support, holding religious and spiritual beliefs, reasons for living, perceived positive personal skills and attributes	Prevent suicidal thoughts and behaviors	N/A
43.	Rodríguez-Quiroga et al. (65), Spain	Quantitative	Adolescents	Suicide risk	Cognitive resilience, interpersonal relationship	Reduce suicide risk	STOP-Suicidality Resilience Factors Scale
44.	Sánchez-Teruel et al. (16), Spain	Quantitative	People who have made a previous suicide attempt	Suicide attempt	Internal protection, external protection, emotional stability	Reduce the high risk of suicide reattempt	Scale of resilience to Suicide Attempts
45.	Kumar et al. (66), USA	Quantitative	Undergraduate students who reported an adolescent or adulthood sexual assault	Posttraumatic stress symptoms linked to sexual assault	Optimism, gratitude	Decrease the adverse impact of a traumatic event, weaken the association between posttraumatic stress and suicidal ideation	N/A
46.	Houchins (67), USA	Dissertation (quantitative)	Active duty U.S. Army Soldiers who reported significant suicidal ideation	Suicide ideation	Reasons for living, positive attributional style, grit, posttraumatic growth, mindfulness, purpose/meaning in life, curiosity, and dispositional optimism	Protect against suicidality	N/A
47.	Bryan et al. (68), USA	Quantitative	US military personnel	High suicide risk	Happiness, meaning in life	Reduce suicide risk	N/A
48.	Fuller-Thomson et al. (69), Canada	Quantitative	Respondents who reported that they had attempted suicide at some point in their lives	Suicide attempt	Having a confidant, being female, older age, higher income, and having no history of mental illness	Free from suicidal thoughts, experience life satisfaction, and psychological wellbeing	N/A
49.	Yu et al. (70), USA	Quantitative	Adolescents with different patterns of depressive symptoms	Depressive symptoms	Life satisfaction, optimism	Buffer against suicidality risk in the face of mild or moderate to severe depressive symptoms	N/A
50.	Richardson et al. (71), UK	Qualitative	Men who had attempted suicide	Suicide attempt	Recognizing the need for help and support, talking, social connections and relationships with others	Manage a crisis, halt the progression from suicidal ideation to behavior	Interview
51.	Ridge et al. (72), UK	Qualitative	Men who self-reported past serious suicidal thinking and intent	Being suicidal	Realizing that they had control of their own fate, gaining an understanding of their distress, initiating meaningful life change, connecting with others, and refocusing on the positive	Move away from suicidal thinking and toward recovery	Interview
52.	Han et al. (9), Australia	Quantitative	Young adults aged between 18 and 25 years who experienced suicidal thoughts in the past year	Being suicidal	Cognitive flexibility, self-efficacy in expressions of positive affect, reduced use of digital technology, less self-harm and substance use for coping	Less severe suicidal thoughts, greater positive affect, and less negative affect	N/A

## Results

The 52 articles included in the analysis were published between 2003 and 2022, and were from the following countries/regions: Australia (*N* = 4), Canada (*N* = 9), Germany (*N* = 1), New Zealand (*N* = 1), Norway (*N* = 1), Spain (*N* = 2), Sweden (*N* = 2), Taiwan (*N* = 5), United States (*N* = 18), United Kingdom (*N* = 9). The study design was quantitative (*N* = 27), qualitative (*N* = 21), and review (*N* = 4). A summary of study information is presented in Table 2.

### Uses of the concept

The first and second steps in the Walker and Avant analysis method have been described in the Introduction section. And the third step in this method is to identify various usages of the concept (27). To achieve this goal, dictionaries and literature were used.

#### Dictionary definitions of suicide resilience

The concept of suicide resilience includes the two sub-concepts of suicide and resilience. Suicide is defined by Merriam-Webster's dictionary as “the act or an instance of taking one's own life voluntarily and intentionally” (73). The Oxford dictionary also defines the word as “the act of killing oneself deliberately” (74). Resilience, another concept, is defined by Merriam-Webster's dictionary as “an ability to recover from or adjust easily to misfortune or change” (75). According to the Oxford dictionary, resilience is “the ability of people or things to recover quickly after something unpleasant” (76). In this broad sense, suicide resilience would be described as an ability to recover from the adversity that may leads individuals to take their life deliberately.

#### Use of suicide resilience in literature

The emergence of the suicide resilience concept is due to the increasing attention on psychological resilience. In psychology, resilience has three inconsistent definitions. It is a personal trait that helps people do well despite adversity, a positive outcome of maintaining mental health despite significant stress or a dynamic process of adapting well to unfavorable circumstances (77, 78). These definitions were applied to the suicide field and greatly affected the concept of suicide resilience.

The term suicide resilience was first used by Osman as the perceived ability, resources, or competence to regulate suicide-related thoughts, feelings, and attitudes (17). Osman also mentioned that suicide resilience incorporated a set of suicide protective factors and divided them into three domains, including internal protective, external protective, and emotional stability. Johnson refers to suicide resilience as positive self-appraisals and suggests that it will buffer against the pernicious impact of stress (34). Later, Johnson defined suicide resilience as a perception or a set of beliefs that buffer individuals from suicidality when facing stressors and developed the buffering hypothesis where suicide resilience factors are not merely protective factors (14). Meanwhile, some researchers directly take the definition of psychological resilience to investigate suicide resilience without giving a specific definition (29, 30).

In the literature, the terms used to describe suicide resilience were also called several other similar terms. Notably, when the type of suicide phrase is a suicide attempt, recovery was found to be the most commonly used word, which can be interpreted as the process definition of suicide resilience (18, 46). Except for suicide resilience, other terms included resilience to suicide, resilience to suicidality, suicidal recovery, overcome suicidality, recovery from suicide, or psychological resilience to suicidal thoughts and behaviors (20, 49).

#### Defining attributes of suicide resilience

The fourth step in the method is to find defining attributes of suicide resilience, that is, the characteristics frequently associated with the concept. By doing so, the concept of suicide resilience can be distinguished from others (27). After an exhaustive screening of the included articles and consultation with the research team, the top five mentioned themes which have significantly higher sum scores than the others were selected as suicide resilience attributes. These attributes are social support, coping strategies, psychological capital, meaning in life, and sense of responsibility.

#### Social support

Social support is a central word in the reviewed literature, with a total of 28 articles mentioned (39, 40, 50, 54, 55, 64). Across studies, individuals who have higher levels of social support are less likely to consider suicide and can recover from being suicidal faster. Social support can come from various sources, including family, partners, friends, relatives, healthcare professionals, community, religion, and pets (43, 46, 59). The social support system for people at high risk of suicide has been identified as a significant influencer on resilient outcomes. Gulbas et al. (56) examined trajectories of wellbeing following a suicide attempt through a longitudinal qualitative research design within 17 Latina teens. They identified three distinct profiles which were labeled as “resilience trajectory” (*N* = 5), “tenuous growth trajectory” (*N* = 6), and “chronic stress trajectory” (*N* = 6). Participants in the “resilience trajectory” categories reported improvements in relationships with family or peers over time, while the other two categories did not report that. Meanwhile, mental health professionals were shown to play a critical role in facilitating the recovery process for participants in every resilient case. Several studies also indicated that receiving support from significant others and professional services was of paramount importance, especially during times of high suicide risk (18, 58).

#### Coping strategies

The second suicide resilience attribute is coping strategies, with a total of 19 articles mentioned (18, 28, 35, 43, 54). Coping strategies refer to the cognitive and behavioral activities individuals engage in to deal with stressful situations encountered in life (79). Positive and healthy coping strategies are inversely related to suicidal ideation and behaviors. Gulbas et al. (56) reported that participants who developed more coping skills were more likely to belong to the resilience groups. Harris et al. (58) reported that talking to people and performing daily activities that demanded a level of concentration (e.g., exercising, listening to music, cooking, and playing computer games) were helpful for patients with schizophrenia diagnoses to suppress their suicidal ideation. Several other research also concluded that emotional expression through talking or writing played an essential role in a suicidal recovery process (29, 71).

#### Psychological capital

A third suicide resilience attribute is psychological capital, which was mentioned in 18 articles (35, 36, 49, 63, 70). Psychological capital is defined as an individual's positive psychological state of development. Initially, it comprises four positive psychological resources: self-efficacy, optimism, hope, and resilience (80). Recently, there is growing discussion to include related nomological constructs such as wellbeing, gratitude, grit, emotional intelligence, mindfulness, and forgiveness into the concept (81). It is selected as an attributes to reflect the broad personal characteristics which are studied frequently in the suicide resilience field. For example, both Kumar et al. (66) and Yu et al. (70) reported that being optimistic could buffer against suicidality risk in the face of PTSD or depressive symptoms. Kleiman et al. (36) reported that participants with a high level of gratitude and grit were characterized with greatest reduction in suicidal ideation over time. Other variables, such as self-efficacy and mindfulness, have been demonstrated to have the same functioning (9, 49).

#### Meaning in life

Meaning in life is linked to suicide resilience with a total of 16 articles mentioned (15, 36, 42, 67, 68). It is a strong protective factor against suicide. Several studies have suggested that individuals with a higher level of meaning in life are less likely to develop suicide ideation with associated risk factors. There is no consistent definition of meaning in life, but all of them share three common features: cognitive component, motivational component, affective component (82). The first component is about making sense of one's experiences in life. Harris et al. (58) and Ridge et al. (72) reported that having an understanding of personal distress and suicidal experience could lead to reconciliation and acceptance of this experience, which was key aspects of establishing suicide resilience. The second component is about the pursuit and attainment of worthwhile goals, and the final component is about feelings of satisfaction, and happiness accompanying goal achievement. Zaheer et al. (59) and Everall et al. (29) reported that creating goals for the future had contributed to the increase of suicide resilience.

#### Sense of responsibility

Sense of responsibility compared to the attributes above is linked to suicide resilience with a total of 7 articles mentioned (29, 30, 54, 58). It is defined as awareness of one's obligation (40). Research demonstrates that sense of responsibility has two types, whether responsibility to others or personal responsibility. Several participants described being worried or realized about the effects their suicidal ideation or behaviors would have on their parents or peers. Chan et al. (43) reported this kind of awareness helped individuals to go on living. In addition, Sun et al. (18) reported the feeling of living for family could contribute to recovery after a suicide attempt. Other participants described that they need to take responsibility for themselves and their health. For example, Tofthagen et al. (48) reported that this awareness leads individuals to quit suicidal behaviors.

#### Cases

According to the Walker and Avant method, several cases are required to further clarify the concept of suicide resilience (27). Here, we present cases that: include all defining attributes (i.e., model cases), contain some but not all of the defining attributes (i.e., borderline cases), and absence of the defining attributes (i.e., contrary cases). The cases were adapted from the literature or constructed by authors.

#### Model case

Collin is a 22-year-old college student. He grew up in a rural family where his father did not talk about emotions. In his early adolescence, he secretly found out that his father had an affair which worsened their relationship. At the time, he also had a distant relationship with his mother and siblings, as well as school friends. Collin felt unhappy and perplexed by his negative emotions. He had suicidal thoughts often and once nearly attempted it. As time went by, he formed a close relationship with a new group of friends and began talking more openly about himself and his experiences (social support, coping strategies). He found that his friends were very supportive and gave him a reason to be excited about life (meaning in life). His relationship with his siblings dramatically improved in his late teen years. His siblings would often choose him to turn to when they needed help or support, which made him feel he had a significant role in the family (sense of responsibility). Collin gradually gained the confidence to accept himself and sees himself as being a strong person who cannot be stopped and who will figure out a way to deal with everything (psychological capital). He is also optimistic about the future, believing that it will bring him success and happiness (meaning in life).

#### Borderline case

Andy is a 36-year-old man who attempted suicide a few years ago. After realizing its negative impact on his family who always support him no matter what, he felt a strong responsibility to live for them. He found a new job as a salesman. He can find a little meaning in the job when helping his customers. However, he felt that he had become a lot more introverted and always had a sense of inferiority after a suicide attempt, which made him have great difficulties in interpersonal communication. Despite his family being supportive, he would not talk to them about his trouble. He found it effective to let out negative emotions through exercising, listening to music, and playing games. Nevertheless, sometimes he would be overwhelmed by the negative feelings and have suicidal thoughts during this tough time.

#### Contrary case

Katie is a 14-year-old middle school student with a diagnosis of depressive disorder. She encountered school bullying and had a hard time going through the experiences. She also has a poor relationship with her parents and almost has no true friends. Her only pleasure in life was her pet cat. She always thought that she had no value in this world and life had no meaning at all. When she felt depressed, she could not find an effective coping strategy to deal with the torturing feeling. She considered suicide as a relief and had attempted suicide by self-poisoning several times. Although she was rescued in every attempt, she still felt suicidal all the time.

#### Antecedents

Antecedents are the events or incidents that lead to the occurrence of the concept. The main antecedent of suicide resilience is the events that may place an individual at high risk of suicide. It may be adversity such as the coming-out process, stressful life events, combat distress, posttraumatic stress symptoms linked to sexual assault or childhood abuse, being in late life, depressive symptoms, and a diagnosis of schizophrenia (28, 34, 42, 44, 50, 64, 66, 67). This adversity also includes events directly relating to suicide, which are the strongest predictors of future suicide, such as suicide ideation or suicide attempt (71, 72).

#### Consequences

Consequences are the events or incidents that occur as a result or outcome of the concept. When individuals demonstrate suicide resilience, outcomes are divided into two types. For those who have not been suicidal yet, suicide resilience enables them to cope effectively with a feeling of despair and buffer them from adversity which heightens suicide risk against the development of suicidal thoughts and behaviors (28, 39, 50). For those who have already been suicidal, suicide resilience can help them overcome suicidality and regain the desire to be alive or transition them from a higher to lower risk of suicide (15, 32, 49). Regardless, the nature of the event is the reduction of suicide risk.

#### Empirical referents

The final step of this analysis is defining the empirical referents which can be used to measure the existence or attributes of the concept. In total, six specific instruments of suicide resilience were identified (16, 17, 34, 47, 61, 65). The detail of each measure's information and involved attributes are shown in Table 3. None of these measures mentioned all defining attributes. Hence, the existing dimensions of them may miss some features of individuals with suicide resilience. Overall, suicide resilience inventory, the suicidal recovery ability scale, and the scale of resilience to suicide attempts cover the most attributes, which suggests they may be more probable measures. Except for suicide resilience inventory and resilience appraisal scale, other scales were all developed in recent years and have not been tested in different cultures.

**Table 3:** Involved defining attributes in six suicide resilience instruments.

**Resilience instruments**	**References**	**Target population**	**Number dimensions (items)**	**Involved defining attributes in six suicide resilience instruments**				
				**Social support**	**Coping strategies**	**Psychological capital**	**Meaning in life**	**Sense of responsibility**
Suicide resilience inventory	Osman et al. (17)	No limit	3 (25)	✓	✓	✓	✓	
Resilience appraisal scale	Johnson et al. (14)	No limit	3 (12)	✓	✓	✓		
The suicide recovery ability scale	Sun et al. (18)	People who have attempted suicide	3 (15)	✓	✓	✓	✓	
Reasons for living-suicide resiliency scale	Wadhwa et al. (61)	Older adults	1 (9)			✓	✓	✓
STOP-Suicidality resilience factors scale	Rodríguez-Quiroga et al. (65)	Child and adolescent	2 (/)	✓		✓		
The scale of resilience to suicide attempts	Sánchez-Teruel et al. (16)	People who have attempted suicide	3 (18)	✓	✓	✓	✓	

## Discussion

**Figure 2:**
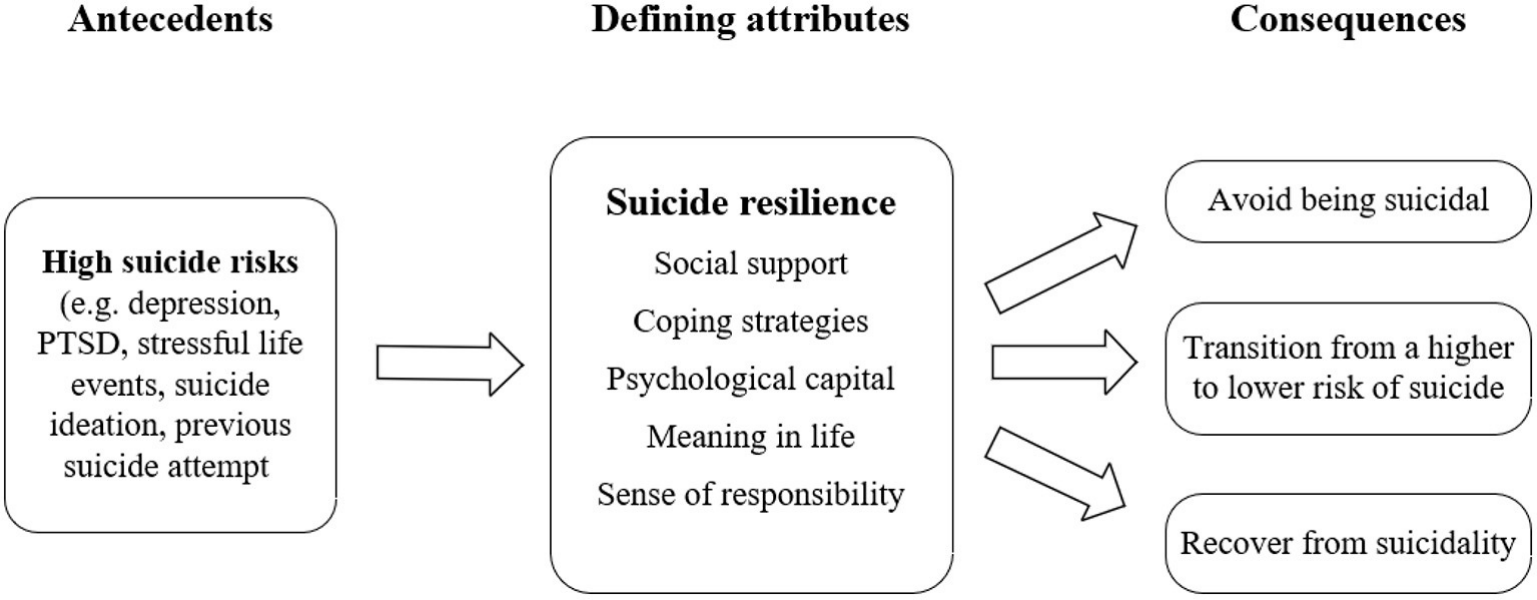
Conceptual model of suicide resilience[[Inline Image]].

In this concept analysis, we identified defining attributes, antecedents, consequences, and empirical references of suicide resilience by applying the Walker and Avant's method, providing a clearer and comprehensive insight into the concept of suicide resilience. Considering all results, we propose the definition of the concept as: suicide resilience is the ability and dynamic process of an individual who is under high suicide risks to avoid being suicidal, transition from a higher to lower risk of suicide, or recover from suicidality, which can be enhanced by the presence of attributes such as social support, coping strategies, psychological capital, meaning in life and sense of responsibility. A conceptual model of suicide resilience was presented in Figure 2.

Our analysis showed that social support, coping strategies, psychological capital, meaning in life, and sense of responsibility were the five attributes of the concept of suicide resilience. The use of case studies further assists in understanding the concept of individuals applying the presence or absence of the attributes in their lives. Some of our results are in line with other studies on resilience. Both social support and coping strategies are commonly mentioned as attributes whether in general or specific resilience research (25, 83, 84). Several positive personalities contained in psychological capital such as optimism and self-efficacy are also popular attributes in similar research (83, 85). However, the other two seem to be specific attributes of suicide resilience.

The existing body of research indicated that events proven to increase suicide risk are the antecedents of suicide resilience. This is in accordance with several other research which reveals that the antecedents of specific resilience are related adversity (86). Harris et al. (58) also demonstrated that individuals can find out whether they have suicide resilience or not unless they have experienced such problems. When encountering suicide-related adversity, individuals with greater suicide resilience levels are more likely to have positive consequences, such as avoiding the development of suicide ideation. Otherwise, they may be trapped in a suicidal circle. Fortunately, more and more studies have revealed that suicide resilience was not a static state, but a dynamic process (18, 58). According to these findings, high level of suicide risks can not only be a challenge but also serve as an opportunity to cultivate or improve suicide resilience.

To prevent suicide risk by enhancing the level of suicide resilience, effective empirical referents are essential. Populations who are vulnerable to suicidality can be identified by using these tools, which can help facilitate the design of early intervention programs. Nevertheless, we only find six scales to measure suicide resilience in literature, and none of them have been widely used. This is consistent with the fact that the concept of suicide resilience has not been widely acknowledged. Based on our analysis, there are no matching measurements that contain all defining attributes. In the future, the existing scales need to be further tested, especially in different cultures, and more suicide resilience scales should be developed. Our suicide resiliency attributes can provide a reference when designing such scales.

This concept analysis has great implications for practice. First, this study informs researchers and clinicians of a growing body of literature on suicide resilience and provides them with a deep understanding of the concept. By doing so, researchers and clinicians can be more aware of the crucial role suicide resilience played in the suicide field, facilitating them to conduct more research on this theme. Second, suicide resiliency attributes identified in this analysis can offer valuable points of intervention to support populations under high suicide risks. Currently, there is rarely explicit or implicit attention to attributes such as meaning in life in mainstream clinical interventions for suicide, which have been found to be useful (36). Hence, more clinical interventions targeting attributes are needed in the future. Third, suicide resilience is defined as a dynamic process which means it will change over time. However, most existing studies on suicide resilience adopt cross-sectional design. Therefore, in order to better understand the concept, more investigation using longitudinal study designs is essential.

Finally, some limitations to this study need to be acknowledged. First, in the selection process of included articles, there might be a risk of bias without quality assessment. Second, we only included articles available in English or Chinese and, therefore, the results might not generalize beyond the settings. Third, suicide resilience research is evolving, which means the results are tentative. It is essential to regularly revisit this analysis to see whether there has anything new.

## Conclusion

This research tried to clarify the concept of suicide resilience and defined it by way of the Walker and Avant concept analysis method. The clarification of the concept may improve researchers' and clinicians' understanding of the nature of suicide resilience, raise their awareness of the importance of assessment, and stimulate more interventions aiming at promoting suicide resilience. Considering that research investigating suicide resilience still use different terms and concepts, which may cause confusion and hinder more research in this area, we hope that this analysis will help to guide future research and interventions by providing a more consistent understanding.

## Author contributions

XW and CD contributed to the design of the study. XW performed the literature research, article selection, data extraction and created the first draft of the manuscript. CD and ZL reviewed and edited the manuscript. All authors contributed to the article and approved the submitted version.

## Conflict of interest

The authors declare that the research was conducted in the absence of any commercial or financial relationships that could be construed as a potential conflict of interest.

## Publisher's note

All claims expressed in this article are solely those of the authors and do not necessarily represent those of their affiliated organizations, or those of the publisher, the editors and the reviewers. Any product that may be evaluated in this article, or claim that may be made by its manufacturer, is not guaranteed or endorsed by the publisher.

## References

[1] Knipe D Padmanathan P Newton-Howes G Chan LF Kapur N. Suicide and self-harm. Lancet. (2022) 399:1903–16. 10.1016/s0140-6736(22)00173-8 35512727

[2] World Health Organization. Suicide Worldwide in 2019. (2021). Available online at: https://www.who.int/publications/i/item/9789240026643 (accessed April 15, 2022).

[3] Nie J O'Neil A Liao B Lu C Aune D Wang Y. Risk factors for completed suicide in the general population: a prospective cohort study of 242, 952 people. J Affect Disord. (2021) 282:707–11. 10.1016/j.jad.2020.12.132 33445097

[4] Bommersbach TJ Rosenheck RA Rhee TG. National trends of mental health care among US adults who attempted suicide in the past 12 months. JAMA Psychiatry. (2022) 79:219–31. 10.1001/jamapsychiatry.2021.3958 35044428 PMC8771432

[5] Cramer RJ Tucker R. Improving the field's understanding of suicide protective factors and translational suicide prevention initiatives. Int J Environ Res Public Health. (2021) 18:1027. 10.3390/ijerph18031027 33503803 PMC7908249

[6] Ivbijaro G Kolkiewicz L Goldberg D Riba MB. N'Jie I NS, Geller J, et al. Preventing suicide, promoting resilience: Is this achievable from a global perspective? Asia Pac Psychiatry. (2019) 11:e12371. 10.1111/appy.12371 31709743

[7] Masten AS. Global perspectives on resilience in children and youth. Child Dev. (2014) 85:6–20. 10.1111/cdev.12205 24341286

[8] Sher L. Resilience as a focus of suicide research and prevention. Acta Psychiatr Scand. (2019) 140:169–80. 10.1111/acps.13059 31150102

[9] Han J Wong I Christensen H Batterham PJ. Resilience to suicidal behavior in young adults: a cross-sectional study. Sci Rep. (2022) 12:11419. 10.1038/s41598-022-15468-0 35794217 PMC9259642

[10] Chang LY Chang YH Wu CC Chang JJ Yen LL Chang HY. Resilience buffers the effects of sleep problems on the trajectory of suicidal ideation from adolescence through young adulthood. Soc Sci Med. (2021) 279:114020. 10.1016/j.socscimed.2021.114020 34004572

[11] Chen X Jiang L Liu Y Ran H Yang R Xu X. Childhood maltreatment and suicidal ideation in Chinese children and adolescents: the mediation of resilience. PeerJ. (2021) 9:e11758. 10.7717/peerj.11758 34277155 PMC8269734

[12] Roy A Sarchiapone M Carli V. Low resilience in suicide attempters: relationship to depressive symptoms. Depress Anxiety. (2007) 24:273–4. 10.1002/da.20265 17120225

[13] Davaasambuu S Aira T Hamid P Wainberg M Witte S. Risk and resilience factors for depression and suicidal ideation in Mongolian college students. Ment Health Prev. (2017) 5:33–9. 10.1016/j.mhp.2017.01.002 28966911 PMC5613944

[14] Johnson J Wood AM Gooding P Taylor PJ Tarrier N. Resilience to suicidality: the buffering hypothesis. Clin Psychol Rev. (2011) 31:563–91. 10.1016/j.cpr.2010.12.007 21276646

[15] Kleiman EM Beaver JKA. meaningful life is worth living: meaning in life as a suicide resiliency factor. Psychiatry Res. (2013) 210:934–9. 10.1016/j.psychres.2013.08.002 23978733

[16] Sánchez-Teruel D Robles-Bello MA Muela-Martínez JA García-León A. Resilience assessment scale for the prediction of suicide reattempt in clinical population. Front Psychol. (2021) 12:673088. 10.3389/fpsyg.2021.673088 34054676 PMC8155352

[17] Osman A Gutierrez PM Muehlenkamp JJ Dix-Richardson F Barrios FX Kopper BA. Suicide resilience inventory-25: development and preliminary psychometric properties. Psychol Rep. (2004) 94:1349–60. 10.2466/pr0.94.3c.1349-1360 15362416

[18] Sun FK Long A. A suicidal recovery theory to guide individuals on their healing and recovering process following a suicide attempt. J Adv Nurs. (2013) 69:2030–40. 10.1111/jan.12070 23294336

[19] Gooding PA Harris K Haddock G. Psychological resilience to suicidal experiences in people with non-affective psychosis: a position paper. Int J Environ Res Public Health. (2022) 19:3813. 10.3390/ijerph19073813 35409502 PMC8997645

[20] Reading L Bowen E. A thematic analysis of how prisoners overcome suicidality. Int J Prison Health. (2014) 10:212–27. 10.1108/ijph-05-2014-0014 25764290

[21] Zhang D Tian Y Wang R Wang L Wang P Su Y. Effectiveness of a resilience-targeted intervention based on “I have, I am, I can” strategy on nursing home older adults' suicidal ideation: a randomized controlled trial. J Affect Disord. (2022) 308:172–80. 10.1016/j.jad.2022.04.046 35439461

[22] Brent D. Prevention programs to augment family and child resilience can have lasting effects on suicidal risk. Suicide Life Threat Behav. (2016) 46 Suppl 1:S39–47. 10.1111/sltb.12257 27094110

[23] Rodgers BL Jacelon CS Knafl KA. Concept analysis and the advance of nursing knowledge: state of the science. J Nurs Scholarsh. (2018) 50:451–9. 10.1111/jnu.12386 29689127

[24] Sun H Yuan C Qian Q He S Luo Q. Digital resilience among individuals in school education settings: a concept analysis based on a scoping review. Front Psychiatry. (2022) 13:858515. 10.3389/fpsyt.2022.858515 35432032 PMC9008236

[25] Van Haeken S Braeken MAKA Nuyts T Franck E Timmermans O Bogaerts A. Perinatal resilience for the first 1,000 days of life. Concept analysis and delphi survey. Front Psychol. (2020) 11:563432. 10.3389/fpsyg.2020.563432 33224056 PMC7670043

[26] Badua AR Caraquel KJ Cruz M Narvaez RA. Vaccine literacy: a concept analysis. Int J Ment Health Nurs. (2022) 31:857–67. 10.1111/inm.12988 35289065 PMC9111838

[27] Walker LO Avant KC. Strategies for Theory Construction in Nursing. Pearson/Prentice Hall Upper Saddle River, NJ (2005).

[28] Fenaughty J Harré N. Life on the seesaw: a qualitative study of suicide resiliency factors for young gay men. J Homosex. (2003) 45:1–22. 10.1300/J082v45n01_01 14567651

[29] Everall RD Altrows KJ Paulson BL. Creating a future: a study of resilience in suicidal female adolescents. J Couns Dev. (2006) 84:461–70. 10.1002/j.1556-6678.2006.tb00430.x35804354

[30] Bostik KE,. Creating a Life Worth Living: A Grounded Theory Investigation of Attachment in Suicidal Adolescents' Process of Healing. (2008). Available online at: https://era.library.ualberta.ca/items/ffbc4dc9-c8c1-45d2-a8c4-5fd169466db3 (accessed March 6, 2022).

[31] Lakeman R FitzGerald M. How people live with or get over being suicidal: a review of qualitative studies. J Adv Nurs. (2008) 64:114–26. 10.1111/j.1365-2648.2008.04773.x 18990092

[32] Bergmans Y Langley J Links P Lavery JV. The perspectives of young adults on recovery from repeated suicide-related behavior. Crisis. (2009) 30:120–7. 10.1027/0227-5910.30.3.120 19767267

[33] Johnson J Gooding PA Wood AM Taylor PJ Pratt D Tarrier N. Resilience to suicidal ideation in psychosis: positive self-appraisals buffer the impact of hopelessness. Behav Res Ther. (2010) 48:883–9. 10.1016/j.brat.2010.05.013 20538264

[34] Johnson J Gooding PA Wood AM Tarrier N. Resilience as positive coping appraisals: Testing the schematic appraisals model of suicide (SAMS). Behav Res Ther. (2010) 48:179–86. 10.1016/j.brat.2009.10.007 19906364

[35] O'Dwyer S Moyle W van Wyk S. Suicidal ideation and resilience in family carers of people with dementia: a pilot qualitative study. Aging Ment Health. (2013) 17:753–60. 10.1080/13607863.2013.789001 23611756

[36] Kleiman EM Adams LM Kashdan TB Riskind JH. Gratitude and grit indirectly reduce risk of suicidal ideations by enhancing meaning in life: evidence for a mediated moderation model. J Res Pers. (2013) 47:539–46. 10.1016/j.jrp.2013.04.007

[37] Benson KM,. Suicide Resilience Among Operation Enduring Freedom Operation Iraqi Freedom Veterans: Sense of Coherence as a Moderator of the Relationship Between Traumatic Experiences Suicidality. Akron: University of Akron (2013). Available online at: http://rave.ohiolink.edu/etdc/view?acc_num=akron1381007889 (accessed March 6, 2022).

[38] Chi MT Long A Jeang SR Ku YC Lu T Sun FK. Healing and recovering after a suicide attempt: a grounded theory study. J Clin Nurs. (2014) 23:1751–9. 10.1111/jocn.12328 24251862

[39] Panagioti M Gooding PA Taylor PJ Tarrier N. Perceived social support buffers the impact of PTSD symptoms on suicidal behavior: implications into suicide resilience research. Compr Psychiatry. (2014) 55:104–12. 10.1016/j.comppsych.2013.06.004 23972619

[40] Kleiman EM Riskind JH Schaefer KE. Social support and positive events as suicide resiliency factors: examination of synergistic buffering effects. Arch Suicide Res. (2014) 18:144–55. 10.1080/13811118.2013.826155 24620940

[41] Matel-Anderson DM Bekhet AK. Resilience in adolescents who survived a suicide attempt from the perspective of registered nurses in inpatient psychiatric facilities. Issues Ment Health Nurs. (2016) 37:839–46. 10.1080/01612840.2016.1193578 27351243

[42] Heisel MJ Flett GL. Does recognition of meaning in life confer resiliency to suicide ideation among community-residing older adults? A longitudinal investigation. Am J Geriatr Psychiatry. (2016) 24:455–66. 10.1016/j.jagp.2015.08.007 26880611

[43] Chan KJ Kirkpatrick H Brasch J. The reasons to go on living project: stories of recovery after a suicide attempt. Qual Res Psychol. (2017) 14:350–73. 10.1080/14780887.2017.1322649

[44] Kapoor S Domingue HK Watson-Singleton NN Are F Elmore CA Crooks CL. Childhood abuse, intrapersonal strength, and suicide resilience in African American females who attempted suicide. J Fam Violence. (2018) 33:53–64. 10.1007/s10896-017-9943-2

[45] Crona L Stenmarker M Öjehagen A Hallberg U Brådvik L. Taking care of oneself by regaining control - a key to continue living four to five decades after a suicide attempt in severe depression. BMC Psychiatry. (2017) 17:69. 10.1186/s12888-017-1223-4 28193192 PMC5307819

[46] Sellin L Asp M Wallsten T Wiklund Gustin L. Reconnecting with oneself while struggling between life and death: the phenomenon of recovery as experienced by persons at risk of suicide. Int J Ment Health Nurs. (2017) 26:200–7. 10.1111/inm.12249 27417106

[47] Sun FK Lu CY Tseng YS Chiang CY. Factors predicting recovery from suicide in attempted suicide patients. J Clin Nurs. (2017) 26:4404–12. 10.1111/jocn.13769 28231627

[48] Tofthagen R Talseth AG Fagerstrøm LM. Former patients' experiences of recovery from self-harm as an individual, prolonged learning process: a phenomenological hermeneutical study. J Adv Nurs. (2017) 73:2306–17. 10.1111/jan.13295 28276577

[49] Collins KRL Stritzke WGK Page AC Brown JD Wylde TJ. Mind full of life: Does mindfulness confer resilience to suicide by increasing zest for life? J Affect Disord. (2018) 226:100–7. 10.1016/j.jad.2017.09.043 28968562

[50] Siegmann P Teismann T Fritsch N Forkmann T Glaesmer H Zhang XC. resilience to suicide ideation: a cross-cultural test of the buffering hypothesis. Clin Psychol Psychother. (2018) 25:e1–9. 10.1002/cpp.2118 28853242

[51] Sun FK Lu CY Huang HM Yu PJ Chiang CY. Development and psychometric testing of the suicidal recovery ability scale (SRAS) for assessing individuals who have attempted suicide. Issues Ment Health Nurs. (2018) 39:954–61. 10.1080/01612840.2018.1463325 30085845

[52] Gallagher ML Miller AB. Suicidal thoughts and behavior in children and adolescents: an ecological model of resilience. Adolesc Res Rev. (2018) 3:123–54. 10.1007/s40894-017-0066-z 29904718 PMC5995470

[53] Roberts, ML,. Adolescent Suicide Prevention: Life Experiences Contributing to Suicidal Ideation Resilience. Ann Arbor: Saybrook University; 2018. Available online at: https://www.proquest.com/dissertations-theses/adolescent-suicide-prevention-life-experiences/docview/2124587637/se-2 (accessed March 6, 2022).

[54] Shaw JL Beans JA Comtois KA Hiratsuka VY. Lived experiences of suicide risk and resilience among Alaska native and American Indian People. Int J Environ Res Public Health. (2019) 16:3953. 10.3390/ijerph16203953 31627325 PMC6843805

[55] Matel-Anderson DM Bekhet AK Garnier-Villarreal M. Mediating effects of positive thinking and social support on suicide resilience. West J Nurs Res. (2019) 41:25–41. 10.1177/0193945918757988 29460692

[56] Gulbas LE Guz S Hausmann-Stabile C Szlyk HS Zayas LH. Trajectories of well-being among Latin adolescents who attempt suicide: a longitudinal qualitative analysis. Qual Health Res. (2019) 29:1766–80. 10.1177/1049732319837541 30920942 PMC6765449

[57] Fullerton L FitzGerald CA Hall ME Green D DeBruyn LM Peñaloza LJ. Suicide attempt resiliency in American Indian, Hispanic, and Anglo Youth in New Mexico: the influence of positive adult relationships. Fam Commun Health. (2019) 42:171–9. 10.1097/fch.0000000000000223 31107727

[58] Harris K Gooding P Haddock G Peters S. Factors that contribute to psychological resilience to suicidal thoughts and behaviours in people with schizophrenia diagnoses: qualitative study. Br J Psych Open. (2019) 5:e79. 10.1192/bjo.2019.63 31496458 PMC6737512

[59] Zaheer J Shera W Sing Hong Lam J Fung WLA Law S Links PS. “I think I am worth it. I can give up committing suicide”: pathways to recovery for Chinese-Canadian women with a history of suicidal behaviour. Transcult Psychiatry. (2019) 56:305–26. 10.1177/1363461518818276 30608027

[60] Fuller-Thomson E Kotchapaw LD. Remission from suicidal ideation among those in chronic pain: what factors are associated with resilience? J Pain. (2019) 20:1048–56. 10.1016/j.jpain.2019.02.096 30979638

[61] Wadhwa S Heisel MJ. Enhancing the assessment of resiliency to suicide ideation among older adults: the development and initial validation of the reasons for living-suicide resiliency scale (RFL-SR). Clin Gerontol. (2020) 43:61–75. 10.1080/07317115.2019.1675840 31635560

[62] Chen X,. Resilience Life Adaptation of Attempted Suicide Adults: Dharma Drum Institute of Liberal Arts. (2020). Available online at: https://www.airitilibrary.com/Publication/alDetailedMesh1?DocID=U0119-0403202014520900 (accessed March 8, 2022).

[63] Clement DN Wingate LR Cole AB O'Keefe VM Hollingsworth DW Davidson CL. The common factors of grit, hope, and optimism differentially influence suicide resilience. Int J Environ Res Public Health. (2020) 17:9588. 10.3390/ijerph17249588 33371423 PMC7767414

[64] Harris K Haddock G Peters S Gooding P. Psychological resilience to suicidal thoughts and behaviours in people with schizophrenia diagnoses: a systematic literature review. Psychol Psychother. (2020) 93:777–809. 10.1111/papt.12255 31625283

[65] Rodríguez-Quiroga A Flamarique I Castro-Fornieles J Lievesley K Buitelaar JK Coghill D. Development and psychometric properties of the “Suicidality: Treatment occurring in paediatrics (STOP) risk and resilience factors scales” in adolescents. Eur Child Adolesc Psychiatry. (2020) 29:153–65. 10.1007/s00787-019-01328-2 31054125 PMC7024696

[66] Kumar SA Jaffe AE Brock RL DiLillo D. Resilience to suicidal ideation among college sexual assault survivors: the protective role of optimism and gratitude in the context of posttraumatic stress. Psychol Trauma Theory Res Pract Policy. (2022) 14:S91–S100. 10.1037/tra0001141 34591537 PMC8930426

[67] Houchins S,. Resilience Suicidality Among Suicidal Soldiers. (2020). Available online at: https://www.proquest.com/openview/99bd411e74350a50da855c9c0b4face2/1?pq-origsite=gscholar&cbl=18750&diss=y (accessed March 6, 2022).

[68] Bryan CJ Bryan AO Kopacz MS. Finding purpose and happiness after recovery from suicide ideation. J Posit Psychol. (2021) 16:46–53. 10.1080/17439760.2019.1676460

[69] Fuller-Thomson E Baiden P Mahoney IP MacNeil A. A bright light at the end of the tunnel: factors associated with complete mental health after a suicide attempt. Arch Suicide Res. (2021) 2021:1–15. 10.1080/13811118.2021.1950088 34313193

[70] Yu J Goldstein RB Haynie DL Luk JW Fairman BJ Patel RA. Resilience factors in the association between depressive symptoms and suicidality. J Adolesc Health. (2021) 69:280–7. 10.1016/j.jadohealth.2020.12.004 33431248 PMC8479833

[71] Richardson C Dickson A Robb KA O'Connor RC. The male experience of suicide attempts and recovery: an interpretative phenomenological analysis. Int J *Environ Res Public Health*. (2021) 18:5209. 10.3390/ijerph18105209 34068854 PMC8153566

[72] Ridge D Smith H Fixsen A Broom A Oliffe J. How men step back—and recover—from suicide attempts: a relational and gendered account. Sociol Health Illn. (2021) 43:238–52. 10.1111/1467-9566.13216 33151571

[73] Suicide. Merriam-Webster's Dictionary. (2022). Available online at: https://www.merriam-webster.com/dictionary/suicide (accessed January 4, 2022).

[74] Suicide. Oxford Learner's Dictionaries. (2022). Available online at: https://www.oxfordlearnersdictionaries.com/us/definition/english/suicide?q=suicide (accessed January 4, 2022).

[75] Resilience. Merriam-Webster's Dictionary. (2022). Available online at: https://www.merriam-webster.com/dictionary/resilience (accessed January 4, 2022).

[76] Resilience. Oxford Learner's Dictionaries. (2022). Available online at: https://www.oxfordlearnersdictionaries.com/us/definition/english/resilience?q=resilience (accessed January 4, 2022).

[77] Zhou Y Ishado E O'Hara A Borson S Sadak T. Developing a unifying model of resilience in dementia caregiving: a scoping review and content analysis. J Appl Gerontol. (2021) 40:1377–88. 10.1177/0733464820923549 32500766

[78] Chmitorz A Kunzler A Helmreich I Tüscher O Kalisch R Kubiak T. Intervention studies to foster resilience—a systematic review and proposal for a resilience framework in future intervention studies. Clin Psychol Rev. (2018) 59:78–100. 10.1016/j.cpr.2017.11.002 29167029

[79] Yi S Chang EC Chang OD Seward NJ McAvoy LB Krause ER. Coping and suicide in college students. Crisis. (2021) 42:5–12. 10.1027/0227-5910/a000662 32238073

[80] Luthans F Youssef CM Avolio BJ. Psychological Capital: Developing the Human Competitive Edge. Oxford: Oxford University Press (2006). 10.1093/acprof:oso/9780195187526.001.0001

[81] Newman A Ucbasaran D Zhu F Hirst G. Psychological capital: a review and synthesis. J Organ Behav. (2014) 35:S120–S38. 10.1002/job.1916

[82] Martela F Steger MF. The three meanings of meaning in life: distinguishing coherence, purpose, and significance. J Posit Psychol. (2016) 11:531–45. 10.1080/17439760.2015.1137623

[83] Garcia-Dia MJ DiNapoli JM Garcia-Ona L Jakubowski R O'Flaherty D. Concept analysis: resilience. Arch Psychiatr Nurs. (2013) 27:264–70. 10.1016/j.apnu.2013.07.003 24238005

[84] Mao X Hu X Loke AY. A concept analysis on disaster resilience in rescue workers: the psychological perspective. Disaster Med Public Health Prep. (2021) 2021:1–10. 10.1017/dmp.2021.157 34286679

[85] Cooper AL Brown JA Rees CS Leslie GD. Nurse resilience: a concept analysis. Int J Ment Health Nurs. (2020) 29:553–75. 10.1111/inm.12721 32227411

[86] Caldeira S Timmins F. Resilience: synthesis of concept analyses and contribution to nursing classifications. Int Nurs Rev. (2016) 63:191–9. 10.1111/inr.12268 27029400

