# Supplementary material for: Suicide resilience: A concept analysis
Source: Front Psychiatry. 2022 Sep 26;13:984922. doi: 10.3389/fpsyt.2022.984922 (PMC9548617; doi:10.3389/fpsyt.2022.984922)
Supplement: Supplementary file 1 [file Table_1.DOCX]

**Strategy for database searches**

| Database | Search terms | Results |
| --- | --- | --- |
| PubMed | (((((((((suicide[MeSH Terms]) OR (suicid*[Title/Abstract])) OR (self-harm[Title/Abstract])) OR (self-injur*[Title/Abstract])) OR (self-destruct*[Title/Abstract])) OR (self-inflict*[Title/Abstract])) OR (self-mutilat*[Title/Abstract])) OR (overdos*[Title/Abstract])) OR (self-poison*[Title/Abstract])) AND ((((((((((resilience, psychological[MeSH Terms]) OR (resilien*[Title/Abstract])) OR (hardiness[Title/Abstract])) OR (buffer*[Title/Abstract])) OR (bounce back[Title/Abstract])) OR (recover*[Title/Abstract])) OR (resist*[Title/Abstract])) OR (rebound[Title/Abstract])) OR (adapt*[Title/Abstract])) OR (overcom*[Title/Abstract])) | 11232 |
| PsycINFO | (TI suicid* OR TI self-harm OR TI self-injur* OR TI self-destruct* OR TI self-inflict* OR TI self-mutilat* OR TI overdos* OR TI self-poison* OR AB suicid* OR AB self-harm OR AB self-injur* OR AB self-destruct* OR AB self-inflict* OR AB self-mutilat* OR AB overdos* OR AB self-poison* OR SU suicid* OR SU self-harm OR SU self-injur* OR SU self-destruct* OR SU self-inflict* OR SU self-mutilat* OR SU overdos* OR SU self-poison* ) AND (TI resilien* OR TI hardiness OR TI buffer* OR TI bounce back OR TI recover* OR TI resist* OR TI rebound OR TI adapt* OR TI overcom* OR AB resilien* OR AB hardiness OR AB buffer* OR AB bounce back OR AB recover* OR AB resist* OR AB rebound OR AB adapt* OR AB overcom* OR SU resilien* OR SU hardiness OR SU buffer* OR SU bounce back OR SU recover* OR SU resist* OR SU rebound OR SU adapt* OR SU overcom*) | 9124 |
| Embase | (suicid*:ab,ti OR 'self harm':ab,ti OR 'self injur*':ab,ti OR 'self destruct*':ab,ti OR 'self inflict*':ab,ti OR 'self mutilat*':ab,ti OR overdos*:ab,ti OR 'self poison*':ab,ti) AND (resilien*:ab,ti OR hardiness:ab,ti OR buffer*:ab,ti OR 'bounce back':ab,ti OR recover*:ab,ti OR resist*:ab,ti OR rebound:ab,ti OR adapt*:ab,ti OR overcom*:ab,ti) | 18302 |
| Web of Science | (TI=(suicid*) OR TI=(self-harm) OR TI=(self-injur*) OR TI=(self-destruct*) OR TI=(self-inflict*) OR TI=(self-mutilat*) OR TI=(overdos*) OR TI=(self-poison*) OR AB=(suicid*) OR AB=(self-harm) OR AB=(self-injur*) OR AB=(self-destruct*) OR AB=(self-inflict*) OR AB=(self-mutilat*) OR AB=(overdos*) OR AB=(self-poison*) OR TS=(suicid*) OR TS=(self-harm) OR TS=(self-injur*) OR TS=(self-destruct*) OR TS=(self-inflict*) OR TS=(self-mutilat*) OR TS=(overdos*) OR TS=(self-poison*)) AND (TI=(resilien*) OR TI=(hardiness) OR TI=(buffer*) OR TI=(bounce back) OR TI=(recover*) OR TI=(resist*) OR TI=(rebound) OR TI=(adapt*) OR TI=(overcom*) OR AB=(resilien*) OR AB=(hardiness) OR AB=(buffer*) OR AB=(bounce back) OR AB=(recover*) OR AB=(resist*) OR AB=(rebound) OR AB=(adapt*) OR AB=(overcom*) OR TS=(resilien*) OR TS=(hardiness) OR TS=(buffer*) OR TS=(bounce back) OR TS=(recover*) OR TS=(resist*) OR TS=(rebound) OR TS=(adapt*) OR TS=(overcom*)) | 20786 |
| CINAHL | (TI suicid* OR TI self-harm OR TI self-injur* OR TI self-destruct* OR TI self-inflict* OR TI self-mutilat* OR TI overdos* OR TI self-poison* OR AB suicid* OR AB self-harm OR AB self-injur* OR AB self-destruct* OR AB self-inflict* OR AB self-mutilat* OR AB overdos* OR AB self-poison* OR SU suicid* OR SU self-harm OR SU self-injur* OR SU self-destruct* OR SU self-inflict* OR SU self-mutilat* OR SU overdos* OR SU self-poison*) AND (TI resilien* OR TI hardiness OR TI buffer* OR TI bounce back OR TI recover* OR TI resist* OR TI rebound OR TI adapt* OR TI overcom* OR AB resilien* OR AB hardiness OR AB buffer* OR AB bounce back OR AB recover* OR AB resist* OR AB rebound OR AB adapt* OR AB overcom* OR SU resilien* OR SU hardiness OR SU buffer* OR SU bounce back OR SU recover* OR SU resist* OR SU rebound OR SU adapt* OR SU overcom*) | 2328 |
| CNKI (China) | (SU %= '自杀' OR SU %= '自伤' OR SU %= '自我伤害' OR SU %= '服毒' OR SU %= '自残' OR TKA = '自杀' OR TKA = '自伤' OR TKA = '自我伤害' OR TKA = '服毒' OR TKA = '自残') AND (SU %= '弹性' OR SU %= '韧性' OR SU %= '抗逆力' OR SU %= '复原力' OR SU %= '恢复' OR SU %= '康复' OR SU %= '抵抗' OR SU %= '克服' OR SU %= '适应' OR SU %= '缓冲' OR TKA = '弹性' OR TKA = '韧性' OR TKA = '抗逆力' OR TKA = '复原力' OR TKA = '恢复' OR TKA = '康复' OR TKA = '抵抗' OR TKA = '克服' OR TKA = '适应' OR TKA = '缓冲') | 4231 |
| WanFang (China) | (主题:(自杀) or 题名:(自杀) or 摘要:(自杀) or 主题:(自伤) or 题名:(自伤) or 摘要:(自伤) or 主题:(自我伤害) or 题名:(自我伤害) or 摘要:(自我伤害) or 主题:(服毒) or 题名:(服毒) or 摘要:(服毒) or 主题:(自残) or 题名:(自残) or 摘要:(自残)) and (主题:(弹性) or 题名:(弹性) or 摘要:(弹性) or 主题:(韧性) or 题名:(韧性) or 摘要:(韧性) or 主题:(抗逆力) or 题名:(抗逆力) or 摘要:(抗逆力) or 主题:(复原力) or 题名:(复原力) or 摘要:(复原力) or 主题:(恢复) or 题名:(恢复) or 摘要:(恢复) or 主题:(康复) or 题名:(康复) or 摘要:(康复) or 主题:(抵抗) or 题名:(抵抗) or 摘要:(抵抗) or 主题:(克服) or 题名:(克服) or 摘要:(克服) or 主题:(适应) or 题名:(适应) or 摘要:(适应) or 主题:(缓冲) or 题名:(缓冲) or 摘要:(缓冲) ) | 4616 |
